# Supplementary material for: A key region of molecular specificity orchestrates unique ephrin-B1 utilization by Cedar virus
Source: Life Sci Alliance. 2019 Dec 20;3(1):e201900578. doi: 10.26508/lsa.201900578 (PMC6925387; doi:10.26508/lsa.201900578)
Supplement: Supplementary file 1 [file LSA-2019-00578_TableS1.docx]

**Supplementary Table 1: Crystallographic data collection and refinement statistics**

| **Data Collection** | **CedV-G** | **CedV-G–ephrin-B1** |
| --- | --- | --- |
| Beamline | DLS I02 | DLS I24 |
| Wavelength (Å) | 0.9795 | 0.9686 |
| Space group | P6_5_ | P2_1_2_1_2_1_ |
| Cell dimensions |  |  |
| *a*, *b*, *c* (Å) | 201.5, 201.5, 112.9 | 112.8, 138.4, 235.4 |
| 𝛼, β, 𝛾 (°) | 90, 90, 120 | 90,90,90 |
| Resolution range (Å) | 58.16-2.78  (2.85-2.78) | 70.19-4.07  (4.14-4.07) |
| R_merge_ | 0.164 (-) | 0.407 (-) |
| R_pim_ | 0.037 (0.412) | 0.173 (0.914) |
| I/σ(I) | 15.4 (2.0) | 3.4 (0.9) |
| CC_1/2_ | 0.998 (0.777) | 0.965 (0.359) |
| Completeness (%) | 99.9 (99.6) | 99.8 (92.4) |
| Multiplicity | 20.5 (19.5) | 6.5 (6.3) |
| Unique reflections | 65,603 (4,849) | 30,120 (1,373) |
| Wilson B-factor | 69.3 | 122.6 |
| **Refinement** |  |  |
| Resolution (Å) | 49.24-2.78 | 69.22-4.07 |
| No. reflections | 65,556 | 30,051 |
| *R*_work_/*R*_free_ | 0.201/0.227 | 0.276/0.314 |
| Protein chains in a.s.u | 2 | 10 |
| No. of atoms | 6,889 | 22,772 |
| Protein/ligand/water | 6,698/182/9 | 22,104/668/0 |
| *B*-factors  Protein/ligand/water | 85.9/102.6/62 | 152.8/175.4/n.a |
| Ramachandran favored/allowed/outlier (%) | 93.9/6.0/0.1 | 92.7/7.2/0.1 |
| Root mean square deviations (RMSD) |  |  |
| Bond lengths (Å) | 0.003 | 0.004 |
| Bond angles (°) | 0.53 | 0.93 |

Values for the highest resolution shell are shown in parentheses.

n.a, non-applicable.
